# First case report of fulminant septic shock from meningococcemia associated with Cryptococcus neoformans coinfection in an immunocompetent patient

**DOI:** 10.1016/j.mmcr.2019.10.003

**Published:** 2019-10-03

**Authors:** Guilherme Dienstmann, Karina Tolfo Avi, Luiz Arthur Calheiros Leite, Joaquim Squizatto Alano, Matheus Leite Ramos de Souza, Marcelo da Silva Mulazani, Paula Cristina Gern Mendivil

**Affiliations:** a Health and Environment Department, University of the Region of Joinville, UNIVILLE, Joinville, SC, Brazil; b Group Ghanem/DASA, Hans Dieter Schmidt Regional Hospital, Joinville, SC, Brazil; c Health Science University of Alagoas State – UNCISAL, Maceió, AL, Brazil; d Internal Medicine Department, Hans Dieter Schmidt Regional Hospital, Joinville, SC, Brazil; e Department of Medicine, University of Region of Joinville – UNIVILLE, Joinville, SC, Brazil; f Infectious Disease Department, Hans Dieter Schmidt Regional Hospital, Joinville, SC, Brazil

**Keywords:** Meningococcemia, Neurocryptococcosis, Coinfection, Immunocompetent

## Abstract

The meningococcal disease manifestation associated with the presence of *Cryptococcus neoformans* is rare. There are no reports in the literature about these simultaneous infections in immunocompetent patients. The aim of the present study is to describe the first case of fulminant septic shock by *Neisseira meningitidis* associated with *Cryptococcus neoformans* coinfection in an immunocompetent patient. We describe a case of an immunocompetent 74-year-old Caucasian woman who presented with fulminant acute meningococcemia associated with cryptococcal meningitis, which progressed to worsening general condition and died of septic shock and multiple organ dysfunctions in less than 48 hours. This case report demonstrates the possibility of coinfections related to *Neisseria meningitidis* and *Cryptococcus neoformans*, even in immunocompetent patients, which represent a diagnostic challenge for clinicians, thus encouraging further studies for a better understanding.

## Highlights

• Meningococcal infections disseminate early, leading to fulminant disease.
• Cryptococcus neoformans meningitis is uncommon in immunocompetent patients.
• The association between meningococcal disease and Cryptococcus neoformans infection is very rare and requires a fast diagnosis and management.
• The real cause of coinfection between Cryptococcus neoformans and Meningococcal disease in immunocompetent patients remains uncertain.

## 1 Introduction

Meningococcemia is an infectious syndrome caused by gram negative diplococci, *Neisseria meningitidis*, a bacterium that is present in the nasopharynx of normal individuals. Meningococcal infection develops when the microorganism spreads from the nasopharyngeal mucosa and invades the bloodstream. Clinical manifestations of meningococcal disease vary, with some mild disease cases, but the most common manifestation is septic syndrome and/or meningitis [1,2].

Infectious meningitis is most often caused by bacteria or viruses. Fungal meningitis is rare, affecting individuals with immunodeficiencies, such as HIV/AIDS, transplanted, and using immunosuppression patients, however this disease is highly dangerous and requires rapid treatment to avoid sequelae [3].

Among the fungal meningitis types, cryptococcal meningitis caused by *Cryptococcus neoformans* is the most common, especially in patients with HIV/AIDS. After entry into the body, the fungus spreads through the bloodstream, reaching the lungs, kidneys, lymph nodes, skin, bones, prostate and ends up being introduced directly into the central nervous system, especially in the meninges. This type of infection is rare in people with a functional immune system and is considered an opportunistic fungus [3]. The immune response and pathogen virulence play an important role in the disease progression and may leads to severe sepsis and, consequently, septic shock, when not immediately treated or inadequately treated [2].

The manifestation of meningococcal disease associated with the presence of *Cryptococcus neoformans* is rare. The present study described the first case of *Nesseria meningitidis* fulminant septic shock associated with *Criptococcus neoformans* coinfection in an immunocompetent patient.

Severe Meningococcal disease (MD) progresses rapidly to shock, multiple organ failure, and death within 24 hours if without urgent treatment. Non-specific symptoms such as fever, drowsiness, nausea and vomiting, irritability and poor appetite are present 4–6 hours after the disease onset. Non-specific sepsis signs, such as pain in the leg, cold hands and feet, and abnormal color, are also observed within 12 hours after disease onset. Classic ecchymotic patches resulting from rapidly developing meningococcal infection and neck pain or stiffness usually appears after 12 hours. Unfortunately, most cases of MD are diagnosed after these late signs onset and it is quite common to find hospitalized patients with an incorrect initial diagnosis [4].

Coagulopathy associated with MD is frequent and usually multifactorial. There is an imbalance between coagulation and fibrinolysis and therefore, although formal coagulation tests may be significantly prolonged, there is a tendency for intravascular thrombosis [5].

The presence of meningococcal endotoxin in the blood generates a severe acute proinflammatory response. Cytokines stimulates the tissue factors release leading to the formation of thrombin and fibrin clots. Cytokines and thrombin inhibit tissue plasminogen activator by releasing the plasminogen activator inhibitor-1 (PAI-1), compromising the endogenous fibrinolytic route. Thrombin formation stimulates inflammatory pathways and further weakens the endogenous fibrinolytic system by activation of the thrombin activatable fibrinolysis inhibitor (TAFI). Activation of the endotoxin complement (mainly via alternative and mannose-binding pathways) leads to the accumulation of anaphylotoxins, such as C3a and C5a that induce endothelial injury [5].

Microthrombosis and endothelial dysfunction associated with the proinflammatory response reduces endothelial expression of thrombomodulin and endothelial protein C receptors, thereby compromising the activation of this protein, disabling fibrinolysis. The procoagulant and proinflammatory state associated with these changes produces endovascular injury, microvascular thrombosis, organ ischemia, and multisystem dysfunction [5].

With inhalation entry port, Cryptococcosis is a systemic mycosis caused by the *Cryptococcus* complex, currently with two species: *Cryptococcus neoformans* and *Cryptococcus gattii*. Both species appears as globular or oval yeast, 3–8 μm in diameter, with single or multiple budding, narrow neck, and surrounded by a characteristic mucopolysaccharides composed capsule [3].

*Cryptococcus neoformans* is cosmopolitan, occurring on various organic substrates, often associated with bird habitat, dry excreta rich in nitrogen sources such as urea and creatinine. Favorable conditions to the abundant growth of this yeast forms microfocus, noted mainly in urban centers and pigeon-related [6]. The home environment, particularly in domestic dust, can be positive, between 13% and 50% [7].

Meningoencephalitis is the most commonly diagnosed clinical form, occurring in more than 80% of cases, either in isolation or associated with pulmonary involvement. It most commonly presents as acute or subacute meningitis or meningoencephalitis, however, single or multiple focal lesions in the central nervous system (CNS), simulating neoplasias, associated or not with the meningeal condition, are observed in the immunocompetent host. This latest presentation has been linked to *Cryptococcus gattii* [8].

In immunocompetent patients the clinical picture, resulting from nervous system inflammation, is exuberant: meningeal signs (nausea, vomiting and stiff neck); signs of meningoencephalitis in one third of patients on admission (changes in consciousness; memory, language and cognition deficit); and involvement of cranial pairs (strabismus, diplopia, or facial paralysis (III, IV, VI and VII) [9].

Temporary or definitive visual impairment or amaurosis throughout the course and treatment reflects injury to the I cranial pair (ophthalmic). There is a great clinical pleomorphism in cryptococcal meningoencephalitis, and dementia may be the only disease manifestation. Physical examination may show meningeal irritation signs (Kerning, Brudzinski, Lasègue, neck stiffness and Lewinson), intracranial hypertension signs, such as papilledema, which usually corresponds to intracranial pressure>350 mmHg. Other neurological signs, such as ataxia, sensory impairment and aphasia may be observed. Complications such as fungal ventriculitis, obstructive block hydrocephalus without meningitis, and cerebrospinal fluid (CSF) malabsorption hydrocephalus by meningitis are frequent [9].

## 2 Case

**Fig. 1:**
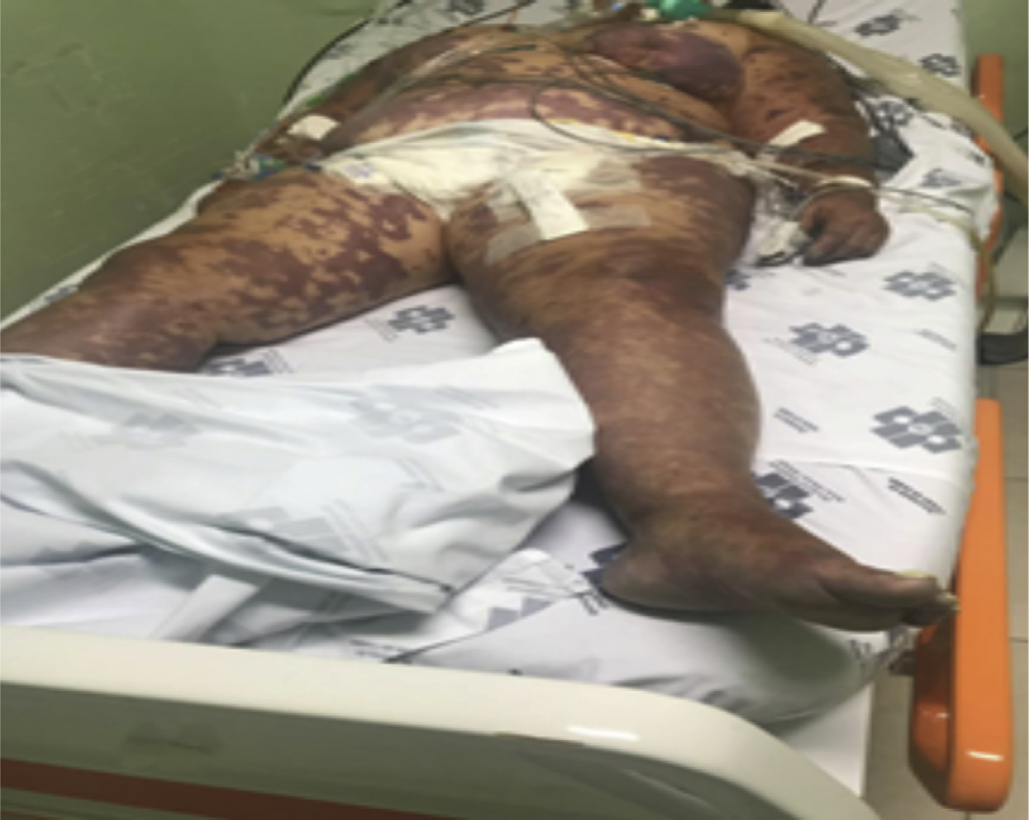
Generalized ecchymotic spots resulting from Meningococcal infection.

Female, 74 years old, living in Londrina (PR), sought emergency care from a neighboring city of Joinville with nausea, vomiting, diarrhea and abdominal pain with 24 hours of evolution, being medicated and released with symptomatics. The patient evolved with consciousness level lowering, oligoanuria, hypotension, cold extremities and generalized ecchymotic spots (Fig. 1). She was then brought to the Emergency Hospital (day 0) in Joinville (SC), in 2019. Family members reported the patient had only systemic arterial hypertension, compensated with monotherapy. She was using losartan and omeprazole. They denied diabetes, alcoholism or other known comorbidities. At physical examination, the patient was unconscious in Glasgow 3, with inaudible blood pressure (BP), tachycardic and tachypneic, and signs of poor peripheral perfusion. Aggressive volume replacement, hemodynamic monitoring, vasoactive drug initiation and subsequent orotracheal intubation, invasive ventilatory support, and respiratory isolation were performed. Laboratory tests showed: Complete blood counts, WBC 30.9 × 109/μL (promyelocytes 1%, myelocytes 2%, metamielocytes 10%, bands 40%, segmented 39%, eosinophils 0%, lymphocytes 5% and monocytes 3%). RBC 3.98 × 1012/μL, hemoglobin 11.7 g/dL, hematocrit 37.4% and platelets counts 56.3 × 109/μL. Albumin 1.6 g/dL (Normal Range NR, 3.5–5.0 g/dL), creatinine 2.29 (NR, 0.52–1.04 mg/dL), potassium 3.3 (NR, 3, 5–5.1 mmol/L), CRP 19.2 (NR, <1.0 mg/dL), lactate 11.6 (NR, 0.5–2.2 mmol/L), prothrombin time/international normatizated ratio (INR) 4.22 (NR, 1.00 to 1.20), activated partial thromboplastin time (ratio) 3.94 (NR, >1.30 seconds). The blood gas analysis presented: pH 6.98 (NR, 7.35 to 7.45); pO2 92.0 (NR, 83.0–108.0 mmHg), pCO2 32.0 (NR, 35.0–48.0 mmHg); HCO3 7.50 (NR, 18.0–23.0 mmol/L); TCO2 8.50 (NR, 22.00–29.00 mmol/L); BE -23.10 (NR, 2.00–3.00 mmol/L) and SO2 91.0% (NR, 95–98%). Cerebrospinal fluid (CSF) examination was cloudy, yellow in color, absent clot, with leucocyte count of 10.9 × 109/μL (polymorphonuclear 85%, lymphomononuclear 15%), red blood cells 8.16 × 109/μL, glucose 29 mg/dL, total proteins 389 mg/dL, chlorides 109 mEq/L, LDH 388 U/L. VDRL and BAAR were negative. Screening and bacterioscopy showed numerous neutrophils and some gram-negative diplococci. The investigation of *cryptococcus* with ink from China showed encapsulated yeast, and the fungal infection by *Cryptococcus neoformans* was confirmed by the research of cryptococcal antigen with latex agglutination test, however it was realized only in CSF. Sorological tests for hepatitis B and C, HIV, CMV and syphilis were negative (day 1). Urine and blood cultures showed no bacterial growth. Genogroup molecular testing (qPCR) for *Neisseria meningitidis* detected genogroup C in CSF and serum sample (day 3). After admission (day 0), ceftriaxone (2g every 12 hours), clindamycin (600mg every 12 hours) and dexamethasone were begun. After fungal infection identification, liposomal amphotericin B (600mg per days), fluconazole (400mg per days) were also begun. The patient evolved (day 2) with refractory septic shock, disseminated intravascular coagulation and progressive hemodynamic instability, without responsiveness to the proposed therapy. Emergency dialyses were attempted without clinical response. The patient evolved to multiple organ dysfunctions and died in less than 48 hours.

## 3 Discussion

The meningococcal disease manifestation associated with the presence of *Cryptococcus neoformans* is rare. There are no reports in the literature about these simultaneous infections in immunocompetent patients.

Inflammatory response related to *Neisseria meningitidis* infection may progress to multiple organ dysfunction, particularly circulatory and renal failure, acute respiratory distress syndrome, and common findings in meningococcal sepsis [10].

Fulminant septic shock associated with meningococcemia causes coagulation disorders, with thrombus formation, and rapid evolution to disseminated intravascular coagulation. Meningococcemia is a life-threatening medical emergency requiring immediate recognition and treatment with antimicrobials and, in some cases, even after appropriate treatment, progresses to death [2].

Meningococcemia usually presents with petechial or purpuric eruption, including mucous membranes, especially in the extremities, and may progress to disseminated eruptions and bruising related to meningococcal septic shock [2]. *Cryptococcus neoformans* is an opportunistic fungus usually associated with immunodeficiency, especially in patients with HIV who are using antiretroviral therapy irregularly. In addition, it may affect patients on prolonged use of corticosteroids, diabetics, Hodgkin's disease, systemic lupus erythematosus, lymphoproliferative diseases, transplantation, sarcoidosis, liver cirrhosis, alcoholism and during chemotherapy [[11], [12], [13]].

In our case, the patient exhibited superficial disseminated purpuric lesions with marked ecchymosis, rapidly evolved into a consuming syndrome, disseminated intravascular coagulation, respiratory failure, and circulatory and renal failure, besides the loss of consciousness, due to a fulminant picture of septic shock, due to meningococcemia, with multiple organ failure and death in less than 48 hours.

We describe the first case of fulminant septic shock due to meningococcemia associated with the presence of *Cryptococcus neoformans* in CSF in an immunocompetent patient. It is noteworthy that the patient had no comorbidity reported in the literature that could lead to infection with *Cryptococcus*, which leads us to the hypothesis of susceptibility to opportunistic fungal infection associated with meningococcemia.

## 4 Conclusion

Meningococcal disease associated with *Cryptococcus neoformans* coinfection is rare. In this case, an immunocompetent patient had acute fulminant meningococcemia associated with neurocriptococcosis, which progressed with general condition worsening and died due to septic shock and multiple organ dysfunctions, in less than 48 hours. This case report highlighted the possibility of coinfections related to *Neisseria meningitidis* and *Cryptococcus neoformans*, even in immunocompetent patients, which represents a diagnostic challenge for clinicians, thus encouraging further studies for a better understanding.

## Declaration of competing interest

There are none.
